# The Added Effect of Artificial Intelligence on Physicians’ Performance in Detecting Thoracic Pathologies on CT and Chest X-ray: A Systematic Review

**DOI:** 10.3390/diagnostics11122206

**Published:** 2021-11-26

**Authors:** Dana Li, Lea Marie Pehrson, Carsten Ammitzbøl Lauridsen, Lea Tøttrup, Marco Fraccaro, Desmond Elliott, Hubert Dariusz Zając, Sune Darkner, Jonathan Frederik Carlsen, Michael Bachmann Nielsen

**Affiliations:** 1 Department of Diagnostic Radiology, Copenhagen University Hospital, Rigshospitalet, 2100 Copenhagen, Denmark; Lea.marie.pehrson@regionh.dk (L.M.P.); Carsten.ammitzboel.lauridsen.01@regionh.dk (C.A.L.); Jonathan.frederik.carlsen@regionh.dk (J.F.C.); Mbn@dadlnet.dk (M.B.N.); 2 Department of Clinical Medicine, University of Copenhagen, 2100 Copenhagen, Denmark; 3 Department of Technology, Faculty of Health and Technology, University College Copenhagen, 2200 Copenhagen, Denmark; 4 Unumed Aps, 1055 Copenhagen, Denmark; Leat@unumed.com (L.T.); Mf@unumed.com (M.F.); 5 Department of Computer Science, University of Copenhagen, 2100 Copenhagen, Denmark; De@di.ku.dk (D.E.); Hdz@di.ku.dk (H.D.Z.); Darkner@di.ku.dk (S.D.)

**Keywords:** artificial intelligence, deep learning, computer-based devices, radiology, thoracic diagnostic imaging, chest X-ray, CT, observer tests, performance

## Abstract

Our systematic review investigated the additional effect of artificial intelligence-based devices on human observers when diagnosing and/or detecting thoracic pathologies using different diagnostic imaging modalities, such as chest X-ray and CT. Peer-reviewed, original research articles from EMBASE, PubMed, Cochrane library, SCOPUS, and Web of Science were retrieved. Included articles were published within the last 20 years and used a device based on artificial intelligence (AI) technology to detect or diagnose pulmonary findings. The AI-based device had to be used in an observer test where the performance of human observers with and without addition of the device was measured as sensitivity, specificity, accuracy, AUC, or time spent on image reading. A total of 38 studies were included for final assessment. The quality assessment tool for diagnostic accuracy studies (QUADAS-2) was used for bias assessment. The average sensitivity increased from 67.8% to 74.6%; specificity from 82.2% to 85.4%; accuracy from 75.4% to 81.7%; and Area Under the ROC Curve (AUC) from 0.75 to 0.80. Generally, a faster reading time was reported when radiologists were aided by AI-based devices. Our systematic review showed that performance generally improved for the physicians when assisted by AI-based devices compared to unaided interpretation.

## 1. Introduction

Artificial intelligence (AI)-based devices have made significant progress in diagnostic imaging segmentation, detection, and disease differentiation, as well as prioritization. AI has emerged as the cutting-edge technology to bring diagnostic imaging into the future [1]. AI may be used as a decision support system, where radiologists reject or accept the algorithm’s diagnostic suggestions, which was investigated in this review, but there is no AI-based device that fully autonomously diagnose or classify findings in radiology yet. Some products have been developed for the purpose of radiological triage [2]. Triage and notification of a certain finding have been a task that has had some autonomy since there is no clinician assigned to re-prioritize the algorithm’s suggestions. Other uses of AI algorithms could be suggestion of treatment options based on disease specific predictive factors [3] and automatic monitoring and overall survival prognostication to aid the physician in deciding the patient’s future treatment plan [4].

The broad application of plain radiography in thoracic imaging and the use of other modalities, such as computed tomography (CT), to delineate abnormalities adds to the number of imaging cases that can provide information to successfully train an AI-algorithm [5]. In addition to providing large quantities of data, chest X-ray is one of the most used imaging modalities. Thoracic imaging has, therefore, not only a potential to provide a large amount of data for developing AI-algorithms successfully, but there is also potential for AI-based devices to be useful in a great number of cases. Because of this, several algorithms in thoracic imaging have been developed—most recently in the diagnosis of COVID-19 [6].

AI has attracted increasing attention in diagnostic imaging research. Most studies demonstrate their AI-algorithm’s diagnostic superiority by separately comparing the algorithm’s diagnostic accuracy to the accuracy achieved by manual reading [7,8]. Nevertheless, several factors seem to prevent AI-based devices from diagnosing pathologies in radiology without human involvement [9], and only few studies conduct observer tests where the algorithm is being used as a second or concurrent reader to radiologists: a scenario closer to a clinical setting [10,11]. Even though diagnostic accuracy of an AI-based device can be evaluated by testing it independently, this may not reflect the true clinical effect of adding AI-based devices, since such testing eliminates the factor of human-machine interaction and final human decision making.

Our systematic review investigated the additional effect AI-based devices had on physicians’ abilities when diagnosing and/or detecting thoracic pathologies using different diagnostic imaging modalities, such as chest X-ray and CT.

## 2. Materials and Methods

### 2.1. Literature Search Strategy

The literature search was completed on 24 March 2021, from 5 databases: EMBASE, PubMed, Cochrane library, SCOPUS, and Web of Science. The search was restricted to peer-reviewed publications of original research written in English from 2001–2021, both years included.

The following specific MESH terms were used in PubMed: “thorax”, “radiography, thoracic”, “lung”, “artificial intelligence”, “deep Learning”, “machine Learning”, “neural networks, computer”, “physicians”, “radiologists”, “workflow”, “physicians”. MESH terms were combined with the following all-fields specific search words and their bended forms: “thorax”, “chest”, “lung”, “AI”, “artificial intelligence”, “deep learning”, “machine learning”, “neural networks”, “computer”, “computer neural networks”, “clinician”, “physician”, “radiologist”, “workflow”.

To perform the EMBASE search, the following combination of text word search and EMTREE terms were used: (“thorax” (EMTREE term) OR “lung” (EMTREE term) OR “chest” OR “lung” OR “thorax”) AND (“artificial intelligence (EMTREE term) OR “machine learning” (EMTREE term) OR “deep learning” (EMTREE term) OR “convolutional neural network” (EMTREE term) OR “artificial neural network” (EMTREE term) OR “ai” OR “artificial intelligence” OR “neural network” OR “deep learning” OR “machine learning”) AND (“radiologist (EMTREE term) OR “ physician” (EMTREE term) OR “clinician” (EMTREE term) OR “workflow” (EMTREE term) OR “radiologist” OR “clinician” OR “physician” OR “workflow”).

We followed the PRISMA guidelines for literature search and study selection. After removal of duplicates, all titles and abstracts retrieved from the search were independently screened by two authors (D.L. and L.M.P.). In case of unresolved disagreements, that could not be determined by consensus vote between D.L. and L.M.P., a third author (J.F.C.) was appointed to assess and resolve the disagreement. Data were extracted by D.L. and L.M.P. using pre-piloted forms. To describe the performance of the radiologists without and with assistance of AI-based devices, we used a combination of narrative synthesis and compared measures of accuracy, area under the ROC curve (AUC), sensitivity, specificity, and time measurements.

For evaluating the risk of bias and assess quality of research, we used the QUADAS-2 tool [12].

### 2.2. Study Inclusion Criteria

Peer-reviewed original research articles published in English, between 2001 and 2021, were reviewed for inclusion. Inclusion criteria were set at follows:

1. AI-based devices, either independent or incorporated into a workflow, used for imaging diagnosis and/or detection of findings in lung tissue, regardless of thoracic imaging modality;
  and
2. an observer test where radiologists or other types of physicians used the AI-algorithm as either a concurrent or a second reader;
  and
3. within the observer test, the specific observer that diagnosed/detected the findings without AI-assistance must also participate as the observer with AI-assistance;
  and
4. outcome measurements of observer tests included either sensitivity, specificity, AUC, accuracy, or some form of time measurement recording observers’ reading time without and with AI-assistance.

Studies where one set of physicians, with the aid of AI, retrospectively re-evaluate another set of physicians’ diagnoses without AI were excluded. AI-based devices that did not detect specific pulmonary tissue findings/pathology, e.g., rib fracture, aneurisms, thyroid enlargements etc. were also excluded.

## 3. Results

**Figure 1:**
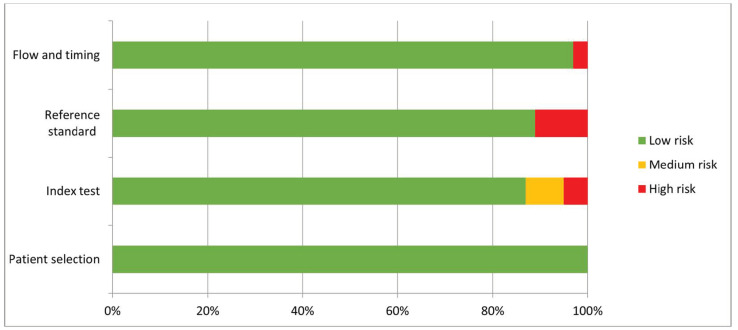
The QUADAS-2 tool for evaluating risk of bias and assess quality of research.

**Figure 2:**
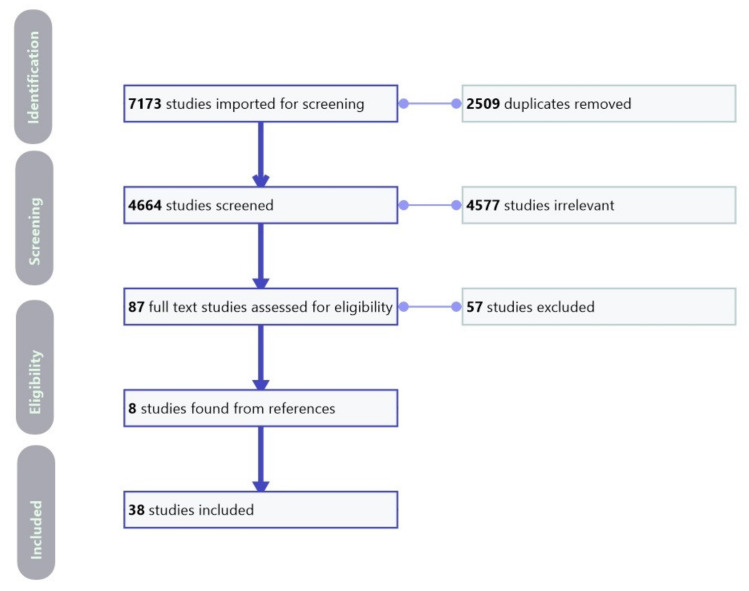
Preferred reporting items for systematic reviews and meta-analyses (PRISMA) flowchart of the literature search and study selection.

We included a total of 38 studies [13,14,15,16,17,18,19,20,21,22,23,24,25,26,27,28,29,30,31,32,33,34,35,36,37,38,39,40,41,42,43,44,45,46,47,48,49,50] in our systematic review. The QUADAS-2 tool is presented in Figure 1, and a PRISMA flowchart of the literature search is presented in Figure 2.

We divided the studies into two groups: The first group, consisting of 19 studies [13,14,15,16,17,18,19,20,21,22,23,24,25,26,27,28,29,30,31], used an AI-based device as a concurrent reader in an observer test, where the observers were tasked with diagnosing images with assistance from an AI-based device, while not being allowed (blinded) to see their initial diagnosis made without assistance from AI (Table 1a). The second group, consisting of 20 studies [19,32,33,34,35,36,37,38,39,40,41,42,43,44,45,46,47,48,49,50] used the AI-based device as a second reader in an un-blinded sequential observer test, thus allowing observers to see and change their original un-assisted diagnosis (Table 1b).

**Figure 3:**
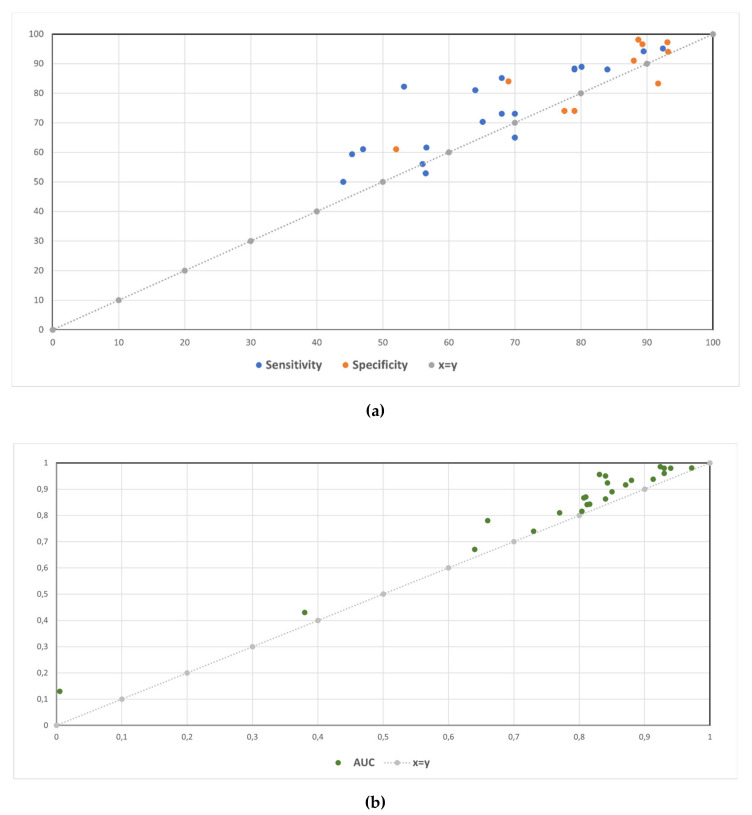
Sensitivity and specificity (**a**) and AUC (**b**) without and with the aid of an AI-based device.

Visual summaries of the performance change in sensitivity, specificity, and AUC for all studies are shown in Figure 3a,b.

### 3.1. Studies Where Human Observers Used AI-Based Devices as Concurrent Readers

In 19 studies observers were first tasked to diagnose the image without an AI-based device. After a washout period, the same observers were then tasked to diagnose the images again. They were not allowed to see and change their original un-aided radiological diagnosis before making their diagnosis aided by and AI-based device (Table 1a). The results of the observer tests are listed in Table 2a–c for concurrent reader studies.

#### 3.1.1. Detection of Pneumonia

Bai et al. [13], Dorr et al. [14], Kim et al. [15] Liu et al. [16], Yang et al. [17], and Zhang et al. [18] had AI-based algorithms to detect pneumonia findings of different kinds, e.g., Covid-19 pneumonia from either non-Covid-19 pneumonia or non-pneumonia. Bai et al. [13], Yang et al. [17], Dorr et al. [14], and Zhang et al. [18] investigated detection of Covid-19 pneumonia. Bai et al. [13], Dorr et al. [14], and Yang et al. [17] all had significant improvement in performance measured in sensitivity after being aided by their AI-based devices (Table 2a), and Zhang et al. [18] reported shorter reading time per image but there was not any mention of statistical significance (Table 2c). Liu et al. [16] incorporated an AI-algorithm into a novel emergency department workflow for Covid-19 evaluations: a clinical quarantine station, where some clinical quarantine stations were equipped with AI-assisted image interpretation, and some did not. They compared the overall median survey time at the clinical quarantine stations in each condition and reported statistically significant shortened time (153 min versus 35 min, *p* < 0.001) when AI-assistance was available. Median survey time specific to the image interpretation part of the clinical quarantine station was also significantly shortened (Table 2c), but they did not report if the shortened reading time were accompanied by the same level of diagnostic accuracy. While the previously mentioned studies specifically investigated Covid-19 pneumonia, Kim et al. [15] used AI-assistance to distinguish pneumonia from non-pneumonia and reported significant improvement in performance measured in sensitivity and specificity after AI-assistance (Table 2a).

##### Detection of Pulmonary Nodules

Beyer et al. [19], de Hoop et al. [20], Koo et al. [21], Kozuka et al. [22], Lee et al. [23], Li et al. [24], Li et al. [25], Liu et al. [26], Martini et al. [27], and Singh et al. [28] used AI-based devices to assist with detection of pulmonary nodules. Even though de Hoop et al. [20] found a slight increase in sensitivity in residents (49% to 51%) and change in radiologists (63% to 61%) for nodule detection, both changes were not statistically significant (Table 2a). In contrast, Koo et al. [21], Li et al. [24], and Li et al. [25] reported improvement of AUC for every individual participating radiologist when using AI-assistance, regardless of experience level (Table 2b). Lee et al. [23] reported improved sensitivity (84% to 88%) when using AI as assistance (Table 2a) but did not mention if the change in sensitivity was significant. However, their reported increase in mean figure of merit (FOM) was statistically significant. Beyer et al. [19] had performed both blinded and un-blinded observer tests; in the blinded, concurrent reader test, radiologists had significant improved sensitivity (56.6% to 61.6%, *p* < 0.001) (Table 2a) but also significantly increased time for reading when assisted by AI (increase of 43 s per image, *p* = 0.04) (Table 2c). Martini et al. [27] reported improved interrater agreement (17–34%) in addition to improved mean reading time (Table 2c), when assisted by AI. Results for the effects of AI assistance on radiologists by Kozuka et al. [22], Liu et al. [26], and Singh et al. [28] are also shown in Table 2a,b, but only Kozuka et al. [22] reported significant improvement (sensitivity from 68% to 85.1%, *p* < 0.01). In addition to change in accuracy, Liu et al. [26] reported a reduction of reading time per patient from 15 min to 5–10 min without mentioning statistical significance.

##### Detection of Several Different Findings and Tuberculosis

Nam et al. [29] tested an AI-based device in detecting 10 different abnormalities and measured the accuracy by dividing them into groups of urgent, critical, and normal findings. Radiologists significantly improved their detection of critical (accuracy from 29.2% to 70.8%, *p* = 0.006), urgent (accuracy from 78.2% to 82.7%, *p* = 0.04), and normal findings (accuracy from 91.4% to 93.8%, *p* = 0.03). Reading times per reading session were only significantly improved for critical (from 3371.0 s to 640.5 s, *p* < 0.001) and urgent findings (from 2127.1 to 1840.3, *p* < 0.001) but significantly prolonged for normal findings (from 2815.4 s to 3267.1 s, *p* < 0.001). Even though Sung et al. [30] showed overall improvement in detection (Table 2a–c), per-lesion sensitivity only improved in residents (79.7% to 86.7%, *p* = 0.006) and board-certified radiologists (83.0% to 91.2%, *p* < 0.001) but not in thoracic radiologists (86.4% to 89.4%, *p* = 0.31). Results from a study by Rajpurkar et al. [31] for the effects of AI-assistance on radiologists detecting tuberculosis show that there were significant improvement in both sensitivity, specificity, and accuracy when aided by AI (Table 2a,b).

### 3.2. Studies Where Human Observers Used AI-Based Devices as a Second Reader in a Sequential Observer Test Design

In 20 studies, observers were first tasked to diagnose the image without an AI-based device. Immediately afterwards, they were tasked to diagnose the images aided by an AI-based device and were also allowed to see and change their initial diagnosis (Table 1b). The results of the observer tests are listed in Table 3a–c for sequential observer test design studies.

#### 3.2.1. Detection of Pulmonary Nodules Using CT

A total of 16 studies investigated the added value of AI on observers in the detection of pulmonary nodules; nine studies [19,32,33,34,35,36,37,38,39] used CT scans, and seven studies [40,41,42,43,44,45,46] used chest X-rays (Table 1b). Although Awai et al. [33], Liu et al. [37], and Matsuki et al. [38] showed statistically significant improvement across all radiologists (Table 3b) when using AI, other studies reported only significant increase in a sub-group of their test observers. Awai et al. [32] and Chen et al. [36] reported only significant improvement in the groups with the more junior radiologists; Awai et al. [32] reported an AUC from 0.768 to 0.901 (*p* = 0.009) in residents but no significant improvement in the board-certified radiologists (AUC 0.768 to 0.901, *p* = 0.19), and Chen et al. [36] reported an AUC from 0.76 to 0.96 (*p* = 0.0005) in the junior radiologists and 0.85 to 0.94 (*p* = 0.014) in the secondary radiologists but no significant improvement in the senior radiologists (AUC 0.91 to 0.96, *p* = 0.221). In concordance, Chae et al. [35] only reported significant improvement in the non-radiologists (AUC from 0.03 to 0.19, *p* < 0.05) but not for the radiologists (AUC from −0.02 to 0.07). While the results from Bogoni et al. [34] confirm the results from Beyer et al.’s [19] concurrent observer test, Beyer et al. [19] showed in the sequential observer test the opposite: decreased sensitivity (56.5 to 52.9, *p* < 0.001) with shortened reading time (294 s to 274 s per image, *p* = 0.04) (Table 3a,c). In addition to overall increase in accuracy (Table 3b), Rao et al. [39] also reported that using AI resulted in greater number of positive actionable management (averaged 24.8 patients), i.e., recommendations for additional images and/or biopsy, that were missed without AI.

#### 3.2.2. Detection of Pulmonary Nodules Using Chest X-ray

As with detection of pulmonary nodules using CT, there were also contrasting results regarding radiologist experience level when using chest X-rays as the test set. Kakeda et al. [41] (AUC 0.924 to 0.986, *p* < 0.001), Kligerman et al. [42] (AUC 0.38 to 0.43, *p* = 0.007), Schalekamp et al. [45] (AUC 0.812 to 0.841, *p* = 0.0001), and Sim et al. [46] (sensitivity 65.1 to 70.3, *p* < 0.001) showed significant improvement across all experience levels when using AI (Table 3a,b). Nam et al. [43] showed significant increase in average among every radiologist experience level (AUC 0.85 to 0.89, *p* < 0.001–0.87), but, individually, there were more observers with significant increase among non-radiologists, residents, and board-certified radiologists than thoracic radiologists. Only one out of four thoracic radiologists had a significant increase. On the other hand, Oda et al. [44] only showed significant improvement for the board-certified radiologists (AUC 0.848 to 0.883, *p* = 0.011) but not for the residents (AUC 0.770 to 0.788, *p* = 0.310). Kasai et al. [40] did not show any statistically significant improvement(Table 3b), but they reported that sensitivity improved when there were only lateral images available (67.9% to 71.6%, *p* = 0.01).

#### 3.2.3. Detection of Several Different Findings

Abe et al. [47], Abe et al. [48], Fukushima et al. [49], and Hwang et al. [50] explored the diagnostic accuracy in detection of several different findings besides pulmonary nodules with their AI-algorithm (Table 1b). While Abe et al. [47] found significant improvement in all radiologists (Table 3b), Fukushima et al. [49] only found significant improvement in the group of radiologists that had more radiological task experience (AUC 0.958 to 0.971, *p* < 0.001). In contrast, Abe et al. [48] found no significant improvement in the more senior radiologists for detection of interstitial disease (*p* > 0.089), and Hwang et al. [50] found no significant improvement in specificity for the detection of different major thoracic diseases in the more senior radiologists (*p* > 0.62). However, there were significant improvements in average among all observers for both studies (Table 3a,b).

## 4. Discussion

The main finding of our systematic review is that human observers assisted by AI-based devices had generally better detection or diagnostic performance using CT and chest X-ray, measured as sensitivity, specificity, accuracy, AUC, or time spent on image reading compared to human observers without AI-assistance.

Some studies suggest that physicians with less radiological task experience benefit more from AI-assistance [30,32,35,36,48,50], while others showed that physicians with greater radiological task experience benefitted the most from AI-assistance [44,49]. Gaube et al. [51] suggested that physicians with less experience were more likely to accept and deploy the suggested advice given to them by AI. They also reported that observers were generally not averse to following advice from AI compared to advice from humans. This suggests that the lack of improvement in the radiologists’ performance with AI-assistance, was not caused by lack of trust in the AI-algorithm but more by the presence of confidence in own abilities. Oda et al. [44] did not find that the group of physicians with less task experience improved from assistance by AI-based device and had two possible explanations. Firstly, the less experienced radiologists had a larger interrater variation of diagnostic performance, leading to insufficient statistical power to show statistical significance. This was also an argument used by Fukushima et al. [49]. Secondly, they argued that the use of AI-assistance lowers false-negative more than false-positive findings, and radiologists with less task experienced generally had more false-positive findings. However, Nam et al. [43] found that physicians with less task experience were more inclined to change their false-negative diagnosis’ and not their false-positive findings; therefore, they benefitted more from AI-assistance. Nam et al. [43], confirmed Oda et al.’s [44] finding in that there was a higher acceptance rate for false-negative findings. Brice [52] also confirmed this and suggested that correcting false-negative findings could have the most impact on reducing errors in radiological diagnosis. Although Oda et al. [44], Nam et al. [43], and Gaube et al. [51] had different reports on which level of physicians could improve their performance the most from the assistance of AI-based devices, they all confirm that AI-assistance lowers false-negative findings, which warrants advancing development and implementation of AI-based devices in to the clinics.

A limitation of our review is the heterogeneity of our included studies, e.g., the different methods for observer testing; some of our studies used a blinded observer test where AI-based devices was used as a concurrent reader (Table 1a), some studies used an un-blinded, sequential observer test (Table 1b), and some used both [19]. To the best of our knowledge, Kobayashi et al. [53] was one of the first to use and discuss both test types. Even though they concluded that there was no statistical significance in the difference of the results obtained from the two methods, they argue that an un-blinded, sequential test type would be less time consuming and practically easier to perform. Since then, others have adopted this method of testing [54] not only in thoracic diagnostic imaging and accepted it as a method for comparing effect of diagnostic tests [55]. Beyer et al. [19] also performed both methods of testing, but they did not come to the same conclusions about the results as Kobayashi et al. [53]. Their results of the two test methods were not the same; In the blinded concurrent reader test, they used more reading time per image (294 s to 337 s, *p* = 0.04) but achieved higher sensitivity (56.5 to 61.6, *p* < 0.001), and, in the un-blinded sequential reader test, they were quicker to interpret each image (294 s to 274 s, *p* = 0.04) but had worse sensitivity (56.5 to 52.9, *p* < 0.001) when assisted by AI. The test observers in the study by Kobayashi et al. [53] did not experience prolonged reading time, even though Bogoni et al. [34] confirmed the results by Beyer et al. [19] and also argued that correcting false-positives would prolong the time spent on an image. Roos et al. [56] also reported prolonged time spent on rejecting false positive cases when testing their computer-aided device and explained that false-positive cases may be harder to distinguish from true-positive cases. This suggests that the sequential observer test design could result in prolonged time spent on reading an image when assisted by a device since they are forced to decide on previous findings. Future observer test studies must, therefore, be aware of this bias, and more studies are needed to investigate this aspect of observer tests.

A pre-requisite for AI-based devices to have a warranted place in diagnostic imaging is that it has higher accuracy than the intended user, since human observers with less experience may have a higher risk of also being influenced by inaccurate advice due to availability bias [57] and premature closure [58]. To be able to include a larger number of studies, we allowed the possibility of some inter-study variability in the performance of the AI-based devices because of different AI-algorithms being used. We recognize this as a limitation adding to the heterogeneity of our systematic review. In addition, we did not review the diagnostic performance of the AI-algorithm by itself, and we did not review the training or test dataset that was used to construct the AI-algorithm. Because of the different AI-algorithms, the included studies may also have been subjected to publication bias since there may be a tendency to only publish well-performing AI-algorithms.

Improved performance in users is a must before implementation can be successful. Our systematic review focused on observer tests performed in highly controlled environments where they were able to adjust their study settings to eliminate biases and variables. However, few prospective clinical trials have been published where AI-based devices have been used, in a more dynamic and clinically realistic environment [59,60]. No clinical trials have been published using AI-based devices on thoracic CT or chest X-rays, whether it be as a stand-alone diagnostic tool or as an additional reader to humans [61]. Our systematic review has, therefore, been a step towards the integration of AI in the clinics by showing that it generally has a positive influence on physicians when used as an additional reader. Further studies are warranted not only on how AI-based devices influence human decision making but also on their performance and integration into a more dynamic, realistic clinical setting.

## 5. Conclusions

Our systematic review showed that sensitivity, specificity, accuracy, AUC, and/or time spent on reading diagnostic images generally improved when using AI-based devices compared to not using them. Disagreements still exist, and more studies are needed to uncover factors that may inhibit an added value by AI-based devices on human decision-making.

## Figures and Tables

**Table 1:** (**a**) Included studies with artificial intelligence-based devices as concurrent readers in the observer test. (**b**) Included studies with artificial intelligence-based devices in an observer test with a sequential test design.

**Author**	**Year**	**Standard of Reference**	**Type of Artificial Intelligence-Based CAD**	**Pathology**	**No. of Cases**	**Test Observers**	**Image Modality**
**a**							
Bai et al. [13]	2021	RT-PCR	EfficientNet-B3 Convolutional Neural Network	COVID-19 pneumonia	119	6 radiologists (10–20 years of chest CT experience)	CT
Beyer et al. [19]	2007	Radiologist identified and consensus vote	Commercially available (LungCAD prototype version, Siemens Corporate Research, Malvern, PA, USA)	Pulmonary nodules	50	4 radiologists (2–11 years experience)	CT
de Hoop et al. [20]	2010	Histologically confirmed	Commercially available (OnGuard 5.0; Riverain Medical, Miamisburg, OH, USA)	Pulmonary nodules	111	1 general radiologist, 1 chest radiologist, and 4 residents	Chest X-ray
Dorr et al. [14]	2020	RT-PCR	DenseNet 121 architecture	COVID-19 pneumonia	60	23 radiologists and 31 emergency care physicians	Chest X-ray
Kim et al. [15]	2020	Bacterial culture and RT-PCR for viruses	Commercially available (Lunit INSIGHT for chest radiography, version 4.7.2; Lunit, Seoul, South Korea)	Pneumonia	387	3 emergency department physicians (6–7 years experience)	Chest X-ray
Koo et al. [21]	2020	Pathologically confirmed	Commercially available (Lunit Insight CXR, ver. 1.00; Lunit, Seoul, South Korea)	Pulmonary nodules	434	2 thoracic radiologists and 2 residents	Chest X-ray
Kozuka et al. [22]	2020	Radiologist identified and majority vote	Faster Region-Convolutional Neural Network	Pulmonary nodules	120	2 radiologists (1–4 years experience)	CT
Lee et al. [23]	2012	Pathologically confirmed	Commercially available (IQQA-Chest, EDDA Technology, Princeton Junction, NJ, USA)	Pulmonary nodules malignant/benign	200	5 chest radiologists and 5 residents	Chest X-ray
Li et al. [24]	2011	CT	Commercially available (SoftView, version 2.0; Riverrain Medical, Miamisburg, OH, USA-Image normalization, feature extraction and regression networks)	Pulmonary nodules	151	3 radiologists (10–25 years experience)	Chest X-ray
Li et al. [25]	2011	Pathologically confirmed and radiology assessed	Commercially available (SoftView, version 2.0; Riverain Medical)	Pulmonary nodules	80	2 chest radiologists, 4 general radiologists, and 4 residents	Chest X-ray
Liu et al. [16]	2020	-	Segmentation model with class attention map including a residual convolutional block	COVID-19 pneumonia	643	-	Chest X-ray
Liu et al. [26]	2019	Radiologist identified and majority vote	DenseNet and Faster Region-Convolutional Neural Network	Pulmonary nodule	271	2 radiologists (10 years experience)	CT
Martini et al. [27]	2021	Radiologist consensus	Commercially available (ClearRead-CT, Riverrain Technologies, Miamisburg, OH, USA)	Pulmonary consolidations/nodules	100	2 senior radiologists, 2 final-year residents, and 2 inexperienced residents	MDCT
Nam et al. [29]	2021	RT-PCR and CT	Deep learning-based algorithm (Deep convolutional neural network)	Pneumonia, pulmonary edema, active tuberculosis, interstitial lung disease, nodule/mass, pleural effusion, acute aortic syndrome, pneumoperitoneum, rib fracture, pneumothorax, mediastinal mass.	202	2 thoracic radiologists, 2 board-certified radiologists, and 2 residents	Chest X-ray
Rajpurkar et al. [31]	2020	Positive culture or Xpert MTB/RIF test	Convolutional Neural Network	Tuberculosis	114	13 physicians (6 months–25 years of experience)	Chest X-ray
Singh et al. [28]	2021	Radiologically reviewed	Commercially available (ClearRead CT Vessel Suppression and Detect, Riverain Technologies TM)	Subsolid nodules (Incl ground-glass and/or part-solid)	123	2 radiologists (5–10 years experience)	CT
Sung et al. [30]	2021	CT and clinical information	Commercially available (Med-Chest X-ray system (version 1.0.0, VUNO, Seoul, South Korea)	Nodules, consolidation, interstitial opacity, pleural effusion, pneumothorax	128	2 thoracic radiologists, 2 board-certified radiologists, 1 radiology resident, and 1 non-radiology resident	Chest X-ray
Yang et al. [17]	2021	RT-PCR	Deep Neural Network	COVID-19 pneumonia	60	3 radiologists (5–20 years experience)	CT
Zhang et al. [18]	2021	RT-PCR	Deep Neural Network using the blur processing method to improve the image enhancement algorithm	COVID-19 pneumonia	15	2 physicians (13–15 years experience)	CT
**Author**	**Year**	**Standard of Reference**	**Type of Artificial Intelligence-Based CAD**	**Pathology**	**No. of Cases**	**Test Observers**	**Image Modality**
**b**							
Abe et al. [47]	2004	Radiological review and clinical correlation	Single three-layer, feed-forward Artificial Neural Network with a back-propagation algorithm	Sarcoidosis, miliary tuberculosis, lymphangitic carcinomatosis, interstitial pulmonary edema, silicosis, scleroderma, P. Carinii pneumonia, Langerhals cell histiocytosis, idiopathic pulmonary fibrosis, viral pneumonia, pulmonary drug toxicity	30	5 radiologists (6–18 years experience)	Chest X-ray
Abe et al. [48]	2003	Radiology consensus	Fourier transformation and Artificial Neural Network	Detection of interstitial lung disease	20	8 chest radiologists, 13 other radiologists, and 7 residents	Chest X-ray
		Clinical correlation and bacteriological	Artificial Neural Network	Differential diagnosis of 11 types of interstitial lung disease	28	16 chest radiologists, 25 other radiologists, and 12 residents	Chest X-ray
		Pathology	Artificial Neural Network	Distinction between malignant and benign pulmonary nodules	40	7 chest radiologists, 14 other radiologists, and 7 residents	Chest X-ray
Awai et al. [33]	2004	Radiological review	Artificial Neural Network	Pulmonary nodules	50	5 board-certified radiologists and 5 residents	CT
Awai et al. [32]	2006	Histology	Neural Network	Pulmonary nodules malignant/benign	33	10 board-certified radiologists and 9 radiology residents	CT
Beyer et al. [19]	2007	Radiologist identified and consensus vote	Commercially available (LungCAD prototype version, Siemens Corporate Research, Malvern, PA, USA)	Pulmonary nodules	50	4 radiologists (2–11 years experience)	CT
Bogoni et al. [34]	2012	Majority of agreement	Commercially available (Lung CAD VC20A, Siemens Healthcare, Malvern, PA, USA)	Pulmonary nodules	43	5 fellowship-trained chest radiologists (1–10 years experience)	CT
Chae et al. [35]	2020	Pathologically confirmed and radiologically reviewed	CT-lungNET (Deep Convolutional Neural Network)	Pulmonary nodules	60	2 medical students, 2 residents, 2 non-radiology physicians, and 2 thoracic radiologists	CT
Chen et al. [36]	2007	Surgery or biopsy	Deep Neural Network	Pulmonary nodules malignant/benign	60	3 junior radiologists, 3 secondary radiologists, and 3 senior radiologists	CT
Fukushima et al. [49]	2004	Pathological, bacteriological and clinical correlation	Single three-layer, feed-forward Artificial Neural Network with a back-propagation algorithm	Sarcoidose, diffuse panbronchioloitis, nonspecific interstitial pneumonia, lymphangitic carcinomatosis, usual interstitial pneumonia, silicosis, BOOP or chronic eopsinophilic pneumonia, pulmonary alveolar proteinosis, miliary tuberculosis, lymphangiomyomatosis, P, carinii pneumonia or cytomegalovirus pneumonia	130	4 chest radiologists and 4 general radiologists	High Resolution CT
Hwang et al. [50]	2019	Pathology, clinical or radiological	Deep Convolutional Neural Network with dense blocks	4 different target diseases (pulmonary malignant neoplasms, tuberculosis, pneumonia, pneumothorax) classified in to binary classification of normal/abnormal	200	5 thoracic radiologists, board-certified radiologists, and 5 non-radiology physicians	Chest X-ray
Kakeda et al. [41]	2004	CT	Commercially available (Trueda, Mitsubishi Space Software, Tokyo, Japan)	Pulmonary nodules	90	4 board-certified radiologists and 4 residents	Chest X-ray
Kasai et al. [40]	2008	CT	Three Artificial Neural Networks	Pulmonary nodules	41	6 chest radiologists and 12 general radiologists	Lateral chest X-ray only
Kligerman et al. [42]	2013	Histology and CT	Commercially available (OnGuard 5.1; Riverain Medical, Miamisburg, OH, USA)	Lung cancer	81	11 board-certified general radiologists (1–24 years experience)	Chest X-ray
Liu et al. [37]	2021	Histology, CT, and biopsy/surgical removal	Convolutional Neural Networks	Pulmonary nodules malignant/benign	879	2 senior chest radiologists, 2 secondary chest radiologists, and 2 junior radiologists	CT
Matsuki et al. [38]	2001	Pathology and radiology	Three-layer, feed-forward Artificial Neural Network with a back-propagation algorithm	Pulmonary nodules	50	4 attending radiologists, 4 radiology fellows, 4 residents	High Resolution CT
Nam et al. [43]	2019	Pathologically confirmed and radiologically reviewed	Deep Convolutional Neural Networks with 25 layers and 8 residual connections	Pulmonary nodules malignant/benign	181	4 thoracic radiologists, 5 board-certified radiologists, 6 residents, and 3 non-radiology physicians	Chest X-ray
Oda et al. [44]	2009	Histology, cytology, and CT	Massive training Artificial Neural Network	Pulmonary nodules	60	7 board-certified radiologists and 5 residents	Chest X-ray
Rao et al. [39]	2007	Consensus and majority vote	LungCAD	Pulmonary nodules	196	17 board-certified radiologists	MDCT
Schalekamp et al. [45]	2014	Radiologically reviewed, pathology and clinical correlation	Commercially available (ClearRead +Detect 5.2; Riverain Technologies and ClearRead Bone Suppression 2.4; Riverain Technologies)	Pulmonary nodules	300	5 radiologists and 3 residents	Chest X-ray
Sim et al. [46]	2020	Biopsy, surgery, CT, and pathology	Commercially available (ALND, version 1.00; Samsung Electronics, Suwon, South Korea)	Cancer nodules	200	5 senior chest radiologists, 4 chest radiologists, and 3 residents	Chest X-ray

**Table 2:** Sensitivity and specificity (**a**); accuracy and AUC (**b**); and time measurement results (**c**) for observer tests without and with AI-based devices as a concurrent reader.

**Author**	**Without AI-Based CAD**		**With AI-Based CAD**		**Change**	**Statistical Significance between Difference**
	**Sensitivity (%)**	**Specificity (%)**	**Sensitivity (%)**	**Specificity (%)**		
**a**						
Bai et al. [13]	79	88	88	91	↑	*p* < 0.001
Beyer et al. [19]	56.5	-	61.6	-	↑	*p* < 0.001
de Hoop et al. [20]	56 *	-	56 *	-	↑	-
Dorr et al. [14]	47	79	61	75	↑	*p* < 0.007
Kim et al. [15]	73.9	88.7	82.2	98.1	↑	*p* < 0.014
Koo et al. [21]	92.4	93.1	95.1	97.2	↑	-
Kozuka et al. [22]	68	91.7	85.1	83.3	↑	*p* < 0.01 **
Lee et al. [23]	84	-	88	-	↑	-
Rajpurkar et al. [31]	70	52	73	61	↑	-
Singh et al. [28]	68 *	77.5 *	73 *	74 *	↑	-
Sung et al. [30]	80.1	89.3	88.9	96.6	↑	*p* < 0.01
Yang et al. [17]	89.5	-	94.2	-	↑	*p* < 0.05
**Author**	**Without AI-Based CAD**		**With AI-Based CAD**		**Change**	**Statistical Significance between Difference**
	**Accuracy (%)**	**AUC**	**Accuracy (%)**	**AUC**		
**b**						
Bai et al. [13]	85	-	90	-	↑	*p* < 0.001
Kim et al. [15]	-	0.871	-	0.916	↑	*p* = 0.002
Koo et al. [21]	-	0.93	-	0.96	↑	*p* < 0.0001
Li et al. [24]	-	0.840	-	0.863	↑	*p* = 0.01
Li et al. [25]	-	0.807	-	0.867	↑	*p* < 0.001
Liu et al. [26]	-	0.66 *	-	0.78 *	↑	-
Nam et al. [29]	66.3 *	-	82.4 *	-	↑	*p* < 0.05
Rajpurkar et al. [31]	60	-	65	-	↑	*p* = 0.002
Singh et al. [28]	-	0.73 *	-	0.74 *	↑	Not statistically significant
Sung et al. [30]	-	0.93	-	0.98	↑	*p* = 0.003
Yang et al. [17]	94.1	-	95.1	-	↑	*p* = 0.01
**Author**	**Without AI-Based CAD**		**With AI-Based CAD**		**Change**	**Statistical Significance between Difference**
	**Time**		**Time**			
**c**						
Beyer et al. [19]	294 s (1)		337 s (1)		↓	*p* = 0.04
Kim et al. [15]	165 min (2)		101 min (2)		↑	-
Kozuka et al. [22]	373 min(2)		331 min (2)		↑	-
Liu et al. [16]	100.5 min (3)		34 min (3)		↑	*p* < 0.01
Liu et al. [26]	15 min (1)		5–10 min (1)		↑	-
Martini et al. [27]	194 s (1)		154 s (1)		↑	*p* < 0.001
Nam et al. [29]	2771.2 s * (1)		1916 s * (1)		↑	*p* < 0.002
Sung et al. [30]	24 s (1)		12 s (1)		↑	*p* < 0.001
Zhang et al. [18]	3.623 min (2)		0.744 min (2)		↑	-

a: * our calculated average; ** for sensitivity only; - not applicable; ↑ positive change. b: * our calculated average; - not applicable; ↑ positive change. c: (1) per image/case reading time; (2) total reading time for multiple cases; (3) station survey time; * our calculated average; - not applicable; ↑ positive change; ↓ negative change.

**Table 3:** Sensitivity and specificity (**a**); accuracy and AUC (**b**); and time measurement results (**c**) for sequential observer tests without and with AI-based devices as a second reader.

**Author**	**Without AI-Based CAD**		**With AI-Based CAD**		**Change**	**Statistical Significance between Difference**
	**Sensitivity (%)**	**Specificity (%)**	**Sensitivity (%)**	**Specificity (%)**		
**a**						
Abe et al. [48]	64	-	81	-	↑	*p* < 0.001
Beyer et al. [19]	56.5	-	52.9	-	↓	*p* < 0.001
Bogoni et al. [34]	45.34 *	-	59.34 *	-	↑	*p* < 0.03
Chae et al. [35]	70 *	69 *	65 *	84 *	↓	Not statistically significant
Hwang et al. [50]	79 *	93.2 *	88.4 *	94 *	↑	*p* = 0.006–0.99
Kligerman et al. [42]	44	-	50	-	↑	*p* < 0.001
Sim et al. [46]	65.1	-	70.3	-	↑	*p* < 0.001
**Author**	**Without AI-Based CAD**		**With AI-Based CAD**		**Change**	**Statistical Significance between Difference**
	**Accuracy (%)**	**AUC**	**Accuracy (%)**	**AUC**		
**b**						
Abe et al. [47]	-	0.81	-	0.87	↑	*p* = 0.031
Abe et al. [48]	-	0.94	-	0.98	↑	*p* < 0.01
Abe et al. [48]	-	0.77	-	0.81	↑	*p* < 0.001
Awai et al. [33]	-	0.64	-	0.67	↑	*p* < 0.01
Awai et al. [32]	-	0.843	-	0.924	↑	*p* = 0.021
Chae et al. [35]	69 *	0.005 *	75 *	0.13 *	↑	Not statistically significant
Chen et al. [36]	-	0.84 *	-	0.95 *	↑	*p* < 0.221
Fukushima et al. [49]	-	0.972 *	-	0.982 *	↑	*p* < 0.071
Hwang et al. [50]	-	0.880 *	-	0.934 *	↑	*p* <0.002
Kakeda et al. [41]	-	0.924	-	0.986	↑	*p* < 0.001
Kasai et al. [40]	-	0.804	-	0.816	↑	Not statistically significant
Kligerman et al. [42]	-	0.38	-	0.43	↑	*p* = 0.007
Liu et al. [37]	-	0.913	-	0.938	↑	*p* = 0.0266
Matsuki et al. [38]	-	0.831	-	0.956	↑	*p* < 0.001
Nam et al. [43]	-	0.85 *	-	0.89 *	↑	*p* < 0.001-0.87
Oda et al. [44]	-	0.816	-	0.843	↑	*p* = 0.011–0.310
Rao et al. [39]	78	-	82.8	-	↑	*p* < 0.001
Schalekamp et al. [45]	-	0.812	-	0.841	↑	*p* = 0.0001
**Author**	**Without AI-Based CAD**		**With AI-Based CAD**		**Change**	**Statistical Significance between Difference**
	**Time**		**Time**			
**c**						
Beyer et al. [19]	294 s (1)		274 s (1)		↑	*p* = 0.04
Bogoni et al. [34]	143 s (1)		225 s (1)		↓	-

a:* our calculated average; - not applicable; ↑ positive change; ↓ negative change. b: * our calculated average; - not applicable; ↑ positive change. c: (1) per image/case reading time; - not applicable; ↑ positive change; ↓ negative change.

## Data Availability

Not applicable.
